# Supplementary material for: Exploring the perception and readiness of Pharmacists towards telepharmacy implementation; a cross sectional analysis
Source: PeerJ. 2022 May 25;10:e13296. doi: 10.7717/peerj.13296 (PMC9147315; doi:10.7717/peerj.13296)
Supplement: Supplemental Information 2 [file peerj-10-13296-s002.pdf]

Exploring Perceptions of Pharmacists towards Telepharmacy Implementation after COVID-19 Pandemic

Section 1 of 5

Dear Pharmacists

We are conducting this survey to explore perceptions of pharmacists towards telepharmacy implementation after the COVID-19 pandemic and the barriers to the implementation of such services in Pakistan. The information provided will be used for research purpose only and the completion of the questionnaire requires only 5 minutes.

Telepharmacy is a method used in pharmacy practice where pharmacist utilizes telecommunications technology to oversee aspects of pharmacy operations or provide remote patient care services. These services include drug monitoring, drug review, Medication Therapy Management, patient assessment and counselling and drug information.

i. **Job Location**

   Hospital pharmacy

   Community pharmacy

ii. **Gender**

   Male

   Female

iii. **Age in years**

iv. **Experience**

   <1

   1-5

   5-10

   >10

v. **Province**

vi. **Education**

   B.Pharm

   Pharm.D

   Master or above

Section 2 of 5

Telepharmacy Implementation for Patients

1). Do you think that telepharmacy will improve the patients' quality of life?

Strongly disagree

Disagree

Neutral

Agree

Strongly agree

2). Do you think that telepharmacy will decrease the patient's visits to hospitals, private clinics or pharmacies?

Strongly disagree

Disagree

Neutral

Agree

Strongly agree

3). Do you think that telepharmacy will decrease the rate of morbidity and mortality in patients?

Strongly Disagree

Disagree

Neutral

Agree

Strongly Agree

4). Do you think that telepharmacy will improve patients' medication adherence?

Strongly Disagree

Disagree

Neutral

Agree

Strongly Agree

5). Do you think that telepharmacy is important to improve patient disease therapy management?

Strongly disagree

Disagree

Neutral

Agree

Strongly agree

6). Do you think that telepharmacy will help in the identification, resolution or prevention of drug-related problems?

Strongly Disagree

Disagree

Neutral

Agree

Strongly Agree

7). Do you think that telepharmacy will help in pharmaceutical care provision by preventing disease transmission?

Strongly Disagree

Disagree

Neutral

Agree

Strongly Agree

8). Do you think that telepharmacy will increase the level of job satisfaction among pharmacists?

Strongly disagree

Disagree

Neutral

Agree

Strongly agree

9). Do you think that telepharmacy will increase patients' appreciation of the pharmacist's role?

Strongly disagree

Disagree

Neutral

Agree

Strongly agree

10). Do you think that telepharmacy will increase job opportunities for pharmacists?

Strongly Disagree

Disagree

Neutral

Agree

Strongly Agree

11). Do you think that telepharmacy is the best choice to reduce pharmacist's burnout rate, especially protection in a pandemic?

Strongly Disagree

Disagree

Neutral

Agree

Strongly Agree

Section 3 of 5

Eligibility

1). Do you think that patients with infectious diseases are the only ones who need telepharmacy services?

Strongly Disagree

Disagree

Neutral

Agree

Strongly Agree

2). Do you think that patients with chronic diseases are the only ones who need telepharmacy services?

Strongly disagree

Disagree

Neutral

Agree

Strongly agree

3). Do you think that geriatric patients using multiple medications are the most eligible group for telepharmacy services?

Strongly Disagree

Disagree

Neutral

Agree

Strongly Agree

Section 4 of 5

Regulatory Issues and Legal Framework for Telepharmacy.

1). Do you think there is a need for a legal collaboration agreement between pharmacists, physicians and other healthcare providers to implement telepharmacy?

Strongly Disagree

Disagree

Neutral

Agree

Strongly Agree

2). Do you think that telepharmacy services should only be provided by a clinical pharmacist?

Strongly Disagree

Disagree

Neutral

Agree

Strongly Agree

3). Do you think that pharmacists should focus on drug-dispensing services only and leave providing telehealth services for physicians?

Strongly Disagree

Disagree

Neutral

Agree

Strongly Agree

4). Do you think that implementing telepharmacy requires facilitating the access of pharmacists to the patients' medical records?

Strongly Disagree

Disagree

Neutral

Agree

Strongly Agree

5). Do you think that implementing telepharmacy requires special tools and space in the pharmacy to communicate with patients and other healthcare providers?

Strongly Disagree

Disagree

Neutral

Agree

Strongly Agree

6). Do you think time limitation could be a barrier to implementing telepharmacy?

Strongly Disagree

Disagree

Neutral

Agree

Strongly Agree

7). Do you think that telepharmacy is an unnecessary work load for pharmacists?

Strongly Disagree

Disagree

Neutral

Agree

Strongly Agree

8). Do you feel more comfortable seeing the patient face-to-face than through telepharmacy?

Strongly Disagree

Disagree

Neutral

Agree

Strongly Agree

9). Do you think that telepharmacy will automate away the social and empathic aspects of care, decreasing its therapeutic value?

Strongly Disagree

Disagree

Neutral

Agree

Strongly Agree

10). Do you think that implementing telepharamcy requires an increase in the number of pharmacists working at different setups?

Strongly Disagree

Disagree

Neutral

Agree

Strongly Agree

11). Do you think that implementing telepharmacy requires special training for pharmacists?

Strongly Disagree

Disagree

Neutral

Agree

Strongly Agree

12). Do you think that implementing telepharmacy requires the availability of adequate drug information services and resources at different setups?

Strongly disagree

Disagree

Neutral

Agree

Strongly agree

13). Do you think that implementing telepharmacy requires a repayment system for the pharmacists who will provide such services?

Strongly Disagree

Disagree

Neutral

Agree

Strongly Agree

14). Do you think that you are prepared to offer telepharmacy services?

Strongly Disagree

Disagree

Neutral

Agree

Strongly Agree

Section 5 of 5

COVID-19 Pandemic and Beyond

1). Do you think that telepharmacy implementation is a good approach during the COVID-19 pandemic and beyond?

Strongly Disagree

Disagree

Neutral

Agree

Strongly Agree

2). Do you think that patients will be interested in receiving telepharmacy services during or after the pandemic?

Strongly Disagree

Disagree

Neutral

Agree

Strongly Agree

3). Do you think that you are familiar with the requirements of telepharmacy implementation?

Strongly Disagree

Disagree

Neutral

Agree

Strongly Agree
